# Efficient Agrobacterium-mediated transformation and genome editing of *Fagopyrum tataricum*

**DOI:** 10.3389/fpls.2023.1270150

**Published:** 2023-09-08

**Authors:** Artur Pinski, Alexander Betekhtin

**Affiliations:** Institute of Biology, Biotechnology and Environmental Protection, Faculty of Natural Sciences, University of Silesia in Katowice, Katowice, Poland

**Keywords:** Agrobacterium-mediated transformation, buckwheat, callus, CRISPR/Cas9 system, Green Fluorescent Protein (GFP), GreenGate system, phytoene desaturase (PDS), β-glucuronidase (GUS)

## Abstract

*Fagopyrum tataricum* (L.) Gaertn. is an exceptional crop known for its remarkable health benefits, high levels of beneficial polyphenols and gluten-free properties, making it highly sought-after as a functional food. Its self-fertilisation capability and adaptability to challenging environments further contribute to its potential as a sustainable agricultural option. To harness its unique traits, genetic transformation in *F. tataricum* is crucial. In this study, we optimised the Agrobacterium-mediated transformation protocol for *F. tataricum* callus, resulting in a transformation rate of regenerated plants of approximately 20%. The protocol’s effectiveness was confirmed through successful GUS staining, GFP expression, and the generation of albino plants via *FtPDS* gene inactivation. These results validate the feasibility of genetic manipulation and highlight the potential for trait enhancement in *F. tataricum*.

## 1 Introduction

*Fagopyrum tataricum*, also known as Tartary buckwheat, is a remarkable crop due to its high content of protein, and beneficial polyphenols, including rutin, quercetin, and other flavonoids, which are lacking in Gramineae crops. These compounds possess antioxidant and anti-inflammatory properties, making Tartary buckwheat an attractive dietary option (Li et al., 2013; Zhou et al., 2018). Moreover, Tartary buckwheat is naturally gluten-free, providing a valuable alternative for individuals with gluten intolerance or celiac disease. These attributes, coupled with its distinct nutty and bitter flavour, contribute to the increasing popularity of Tartary buckwheat as a functional food with health benefits (Noreen et al., 2021). In comparison to *Fagopyrum esculentum*, which is a commonly cultivated crop, *F. tataricum* holds an advantage as it is self-pollinating, eliminating the need for reliance on external pollinators (Yasui et al., 2016). *F. tataricum* is adaptable to challenging environmental conditions, including high-altitude regions characterised by intense ultraviolet radiation. It can thrive in poor soils, making it a promising crop option for areas with limited agricultural suitability contributing to diversification in impoverished areas. Furthermore, cultivating *F. tataricum* can contribute to the preservation of traditional farming systems and the conservation of biodiversity (Kreft et al., 2022; Martínez-Goñi et al., 2023).

Efforts have been made to explore the potential of buckwheat as a functional food, facilitated by the availability of high-quality genomes (Zhang et al., 2017; He et al., 2023; Lin et al., 2023). Recently, Zhang et al. (2021a) developed a comprehensive database based on whole-genome resequencing of 510 germplasms uncovering the genetic diversity present in Tartary buckwheat. Multi-omic analysis comparing *F. esculentum* and *F. tataricum* has shed light on the genetic mechanisms involved in flavonoid biosynthesis and self-incompatibility, opening up opportunities for genetic manipulation and targeted breeding of Tartary buckwheat (He et al., 2023; Lin et al., 2023).

Agrobacterium-mediated transformation is a valuable technique in plant genetic engineering, as it enables the delivery and integration of foreign DNA into the plant genome. This technique utilises the natural ability of *Agrobacterium tumefaciens*, a soil bacterium, to transfer a portion of its DNA (T-DNA) into the plant cells it infects (Song et al., 2019; Pratiwi and Surya, 2020). The T-DNA can be customized to deliver and integrate into plant cells various constructs. The introduction of the Golden Gate cloning system, including its plant-specific variant, the GreenGate system, has revolutionised molecular biology by offering a versatile platform for DNA construct customisation and simplified vector preparation (Lampropoulos et al., 2013). By employing type IIS restriction enzymes, such as BsaI, and a predefined set of genetic modules, the GreenGate system allows for the seamless assembly of DNA constructs with various genetic elements, including promoters, coding sequences, tags, terminators, and resistance cassettes. This system not only facilitates the construction of generic vectors expressing *GUS* (β-glucuronidase) or *GFP* but also enables the development of vectors for targeted mutagenesis using the CRISPR/Cas9 system (Decaestecker et al., 2019). The CRISPR/Cas9 system utilizes a Cas9 protein, guided by a single-guide RNA (sgRNA), to target specific genomic sequences as a programmable DNA endonuclease. Upon binding, Cas9 introduces double-strand breaks (DSBs) at the target site, and the predominant repair pathway in plants, non-homologous end joining (NHEJ), often leads to the insertion or deletion of nucleotides (indels). This indel formation can result in gene knockout or disruption, allowing for precise and targeted mutagenesis (Son and Park, 2022).

Using reporter genes such as GUS and GFP in plant transformation protocols has proven invaluable for optimising experimental procedures. *GUS* and *GFP* genes offer distinct advantages in monitoring and evaluating the success and efficiency of plant transformation events. GUS staining enables reliable detection through the hydrolysis of X-Gluc compound (5-bromo-4-chloro-3-indolyl-β-D-glucuronide) by the GUS enzyme, leading to the production of colourless glucuronic acid and an intense blue precipitate of chloro-bromoindigo. In contrast, GFP enables real-time visualisation of expression patterns on live material (Murray et al., 2004; Serganova and Blasberg, 2019). Fusing the GFP gene with a gene of interest or incorporating sorting peptides allows for easy tracking of the expression and localization of the fusion protein (Berthold et al., 2019).

The inactivation of the *PHYTOENE DESATURASE* (*PDS*) gene is frequently employed as a target for testing the activity of the CRISPR/Cas9 system in plants (Hus et al., 2020). The *PDS* gene is a suitable choice due to its well-characterised function in the carotenoid biosynthesis pathway, being crucial for synthesising pigments responsible for green colouration in plants. By disrupting the *PDS* gene, the production of carotenoids is hindered, leading to the accumulation of phytoene, a colourless precursor, and resulting in the albino phenotype. The phenotypic changes resulting from *PDS* gene disruption enable a visual and cost-effective evaluation of CRISPR/Cas9-mediated gene editing at an early stage, often in regenerated plants or callus tissue. Further, the inactivation of the *PDS* gene is a recessive trait, requiring mutations in both alleles to produce the albino phenotype. Once the gene is disrupted, the lack of functional PDS enzyme activity prevents the recovery of the green colouration in subsequent growth stages (Banfalvi et al., 2020; Komatsu et al., 2020).

Establishing and refining an Agrobacterium-mediated transformation protocol for Tartary buckwheat is crucial to fully harness its potential (Pinski et al., 2023). Till now, successful transformations in *F. tataricum* have primarily focused on establishing hairy root cultures using *Agrobacterium rhizogens* and transforming Tartary buckwheat calli without plants regeneration (Liu et al., 2013; Thwe et al., 2016; Mi et al., 2020; Tomasiak et al., 2022; Wen et al., 2022). The goal of this work was to establish an efficient transformation protocol for *F. tataricum* using Agrobacterium-mediated transformation, proved by the successful incorporation and expression of *GUS* and *GFP* reporter genes. The study aimed to demonstrate the feasibility of utilizing the CRISPR/Cas9 system in *F. tataricum* by targeting and inactivating *PDS* gene, resulting in the production of albino plants.

## 2 Results

### 2.1 A stable and efficient genetic transformation system for *F. tataricum*

**Figure 1:**
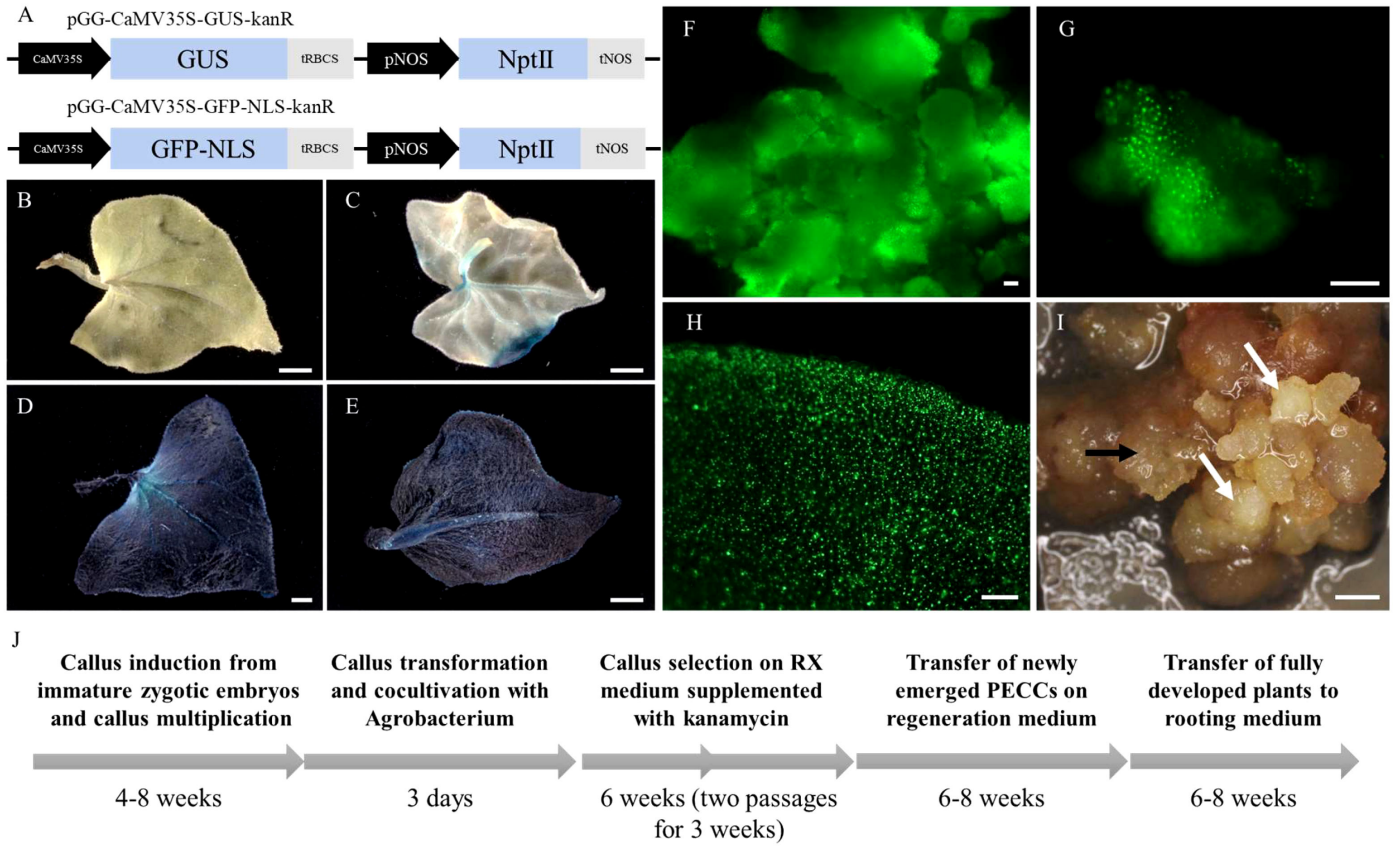
Visualization of GUS staining and GFP fluorescence in callus and plants of *F. tataricum*. **(A)** Schematic representation of GFP (pGG-CaMV35S-GFP-NLS-kanR) and GUS (pGG-CaMV35S-GUS-kanR) expression vectors. **(B)** Leaves of wild-type (WT) regenerated plant of *F. tataricum*. **(C)** Regenerated plant exhibiting mosaicism. **(D-E)** Leaves of transformed plants of *F. tataricum*. **(F-G)** Callus expressing GFP. **(H)** Leaves of regenerated plants expressing GFP. **(I)** Morphogenic callus of *F. tataricum* four weeks after transformation (second passage on selecting medium). The white arrows point at appearing PECCs, while the black arrows indicate a ‘soft’ callus. **(J)** Flowchart illustrating the time frame for *F. tataricum* transformation. Scale bars: 1 mm **(A-D)**, 100 µm **(F-I)**.

We optimised the Agrobacterium-mediated transformation protocol of callus to establish a robust and effective genetic transformation system in *F. tataricum*. Previous studies have demonstrated the successful induction of morphogenic calli from immature embryos, characterised by high morphological and genetic stability, and regeneration potential, making it an ideal target for transformation (Betekhtin et al., 2017; Betekhtin et al., 2019). Transformation efficiency was evaluated using *A. tumefaciens* GV3101 carrying a GUS expression vector assembled using GreenGate parts (pGG-CaMV35S-GUS-kanR) (**Figure 1A**). GUS staining was performed on leaves of regenerated plants to assess the transformation efficiency. Out of the total 119 plants obtained from multiple transformation rounds, 24 plants exhibited blue precipitation indicating GUS enzyme activity, resulting in a transformation efficiency rate of 20.2% (**Figures 1B-D**; **Table 1**; **Supplementary Figure S1**). Some of the regenerated plants displayed mosaicism (8 plants, 6.7%), with only certain parts of the leaves showing successful transformation and GUS expression (**Figure 1C**). Additionally, we introduced a GFP expression vector (pGG-CaMV35S-GFP-NLS-kanR) carrying GFP with nucleolar localisation sequences (NLS) into the callus using Agrobacterium cells. Microscopic examination confirmed the efficient expression of GFP and its nuclear targeting in the callus and regenerated plants (**Figures 1F–H**).

**Table 1:** The efficiency of transformation of *F. tataricum* with pGG-CaMV35S-GUS-kanR vector.

Observed phenotype	Wild-type	GUS-expressing plants	Mosaic plants	Total number of regenerated plants
Number of plants	87	24	8	119
% of all regenerated plants	73.1%	20.2%	6.7%	100%

The transformation of the morphogenic callus was performed using a callus in the middle of the passage, which exhibited visible proembryogenic cell complexes (PECCs) on its surface. The morphogenic callus is comprised of both ‘soft’ callus and PECCs. During the cyclic development of the callus, the mature PECCs undergo disintegration. PECCs contain subsurficial meristematic cells, the source of embryogenically determined cells from which new PECCs arise (Betekhtin et al., 2017). However, for the transformation process, the entire callus containing both PECCs and ‘soft’ callus cells was utilized. Typically, it takes approximately 4-6 weeks after transformation for the young PECCs to become visible on the callus surface (**Figure 1I**). Since the PECCs have the ability to regenerate into plants, only they are transferred to the regeneration medium. A detailed workflow outlining the steps of callus induction, transformation, and regeneration can be found in **Figure 1J**.

### 2.2 Targeted inactivation of *FtPDS* gene using CRISPR/Cas9 system

**Figure 2:**
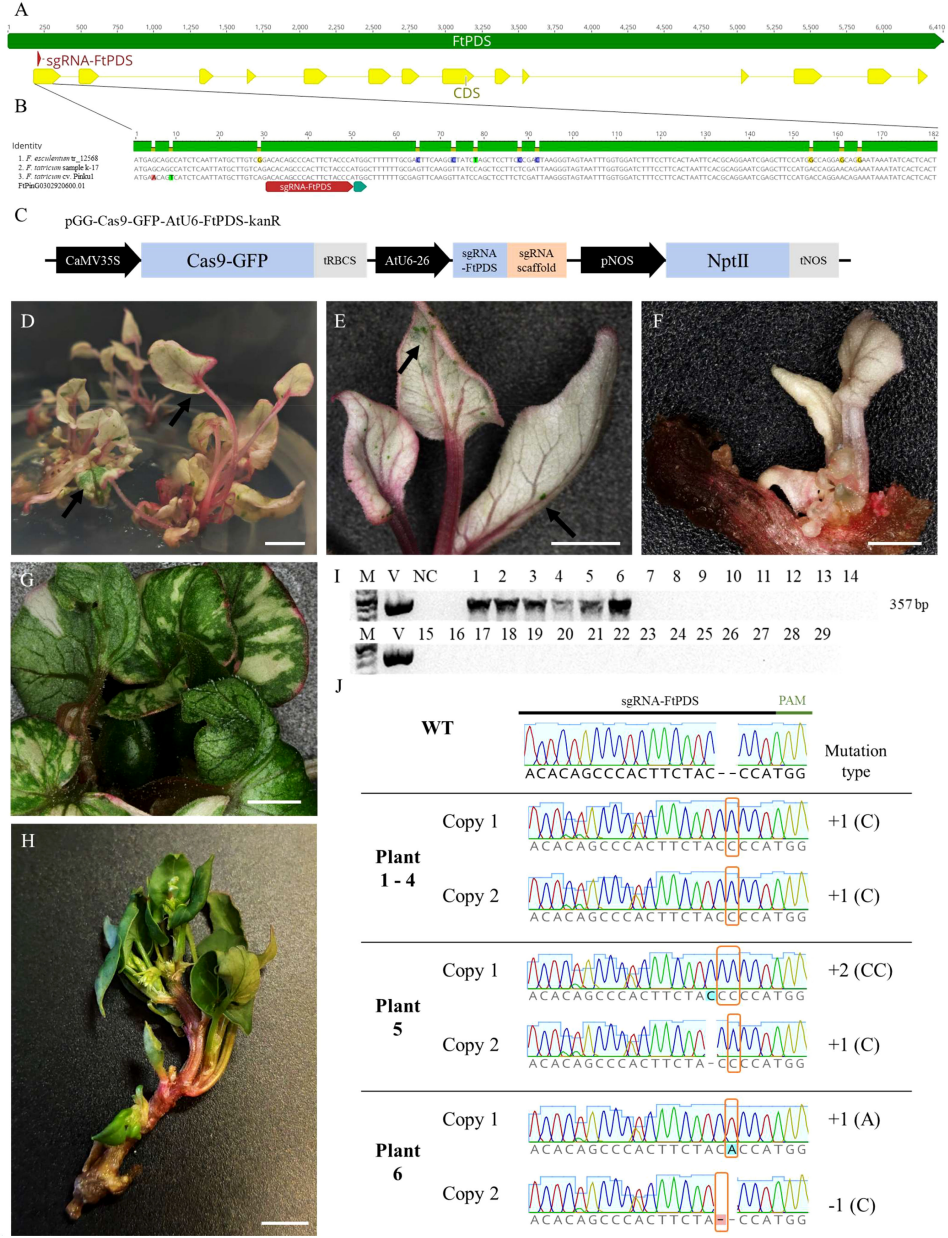
CRISPR/Cas9-mediated targeted mutagenesis of the *FtPDS* gene in *F. tataricum*. **(A)** Schematic representation of the *FtPDS* gene highlighting the sequence targeted by the sgRNA-FtPDS. **(B)** The first exon of the *PDS* gene was aligned among *F. esculentum* cv. DASHA, *F. tataricum* sample k-17, and *F. tataricum* cv. Pinku1. **(C)** Schematic representation of the CRISPR/Cas9 vector pGG-Cas9-GFP-AtU6-FtPDS-kanR. **(D, E)** The regenerate albino growing in the rooting medium. On some leaves, small greenish spots can be observed as indicated by black arrows. **(F)** Regenerated albino plant with biallelic mutation in the *FtPDS* gene. **(G)** Regenerated plant exhibiting mosaicism. **(H)** The regenerated wild-type plant. **(I)** PCR amplification and detection of CRISPR/Cas9 T-DNA integration in the genome of *F. tataricum* plants. M – 1 kb Plus DNA Ladder, V – vector pGG-Cas9-GFP-AtU6-FtPDS-kanR, 1-6 – albino plants, 8-29 wild type plants, NC – negative control. **(J)** The chromatograms with relevant description of detected mutations in the *FtPDS* gene in regenerated plants. The indels in *pds* mutants were indicated by orange frame. Scale bars: 5 mm.

Despite the unavailability of genomic data for the k-17 sample of *F. tataricum* used in this study, we successfully designed primers based on the genomic sequence of *F. tataricum* cv. Pinku1 for amplifying a partial sequence of the *PDS* gene. The obtained sequence was utilised for designing a specific sgRNA targeting the first exon of the *FtPDS* gene (**Figure 2A**). The selected sgRNA sequence (5’-ACACAGCCCACTTCTACCCA-3’) exhibited high specificity with no off-target sites and demonstrated a predicted activity score of 0.305 and a specificity score of 100%. To assess the potential broader applicability of the designed sgRNA targeting the *PDS* gene, we aligned the partial PDS sequence obtained from the k-17 sample with the available genomic sequences of *F. tataricum* and *F. esculentum*. The alignment revealed high similarity in the coding region (**Figure 2B**). This indicates that the designed sgRNA can potentially be utilised for other *F. tataricum* cultivars and *F. esculentum*. The primers, incorporating the specific overlap sequence and *FtPDS*-targeting sgRNA, were assembled and introduced into the AarI-digested vector pGG-Cas9-GFP-AtU6-lacZ-kanR (**Figure 2C**). The resulting vector, pGG-Cas9-GFP-AtU6-FtPDS-kanR, was identified through blue-white screening, confirmed by sequencing, and introduced to Agrobacterium cell that were used for transformation. Out of 30 regenerated plants, six plants displayed an albino phenotype, indicating the potential inactivation of both copies of the *FtPDS* gene (**Figures 2D-F**). This corresponded to a mutation of both copies efficiency rate of 20% (**Table 2**). In one plant, we have observed mosaicism (**Figure 2G**). The PCR reaction amplifying the part of Cas9 gene sequence showed its presence in six albino plant, while being absent in the wild-type plants (**Figure 2I**). To confirm the inactivation of the *FtPDS* gene, we amplified the *FtPDS* gene region, cloned it into the pGEM vector, and performed sequencing analysis (**Figure 2J**). The original sequencing data are presented in the **Supplementary Data 1**. The sequencing results revealed the presence of single or double nucleotide indels in the expected region targeted by the designed sgRNA. These introduced mutations caused a frame shift in the reading frame, resulting in a premature stop codon and subsequent inactivation of the PDS enzyme. The *pds* mutants showed diminished growth rates comparing to the wild-type (**Figures 2D-F, H**). Notably, the mutants displayed distinct pink pigmentation in their petioles and leaf veins (**Figures 2D-F**). This phenotypic change could be attributed to the accumulation of anthocyanins, colourful compounds abundantly found in Fagopyrum species (Fang et al., 2019). In the wild-type, the pink colour can be observed in the stalks of the plants (**Figure 2H**) but not in the petioles and leaf veins, possibly due to the presence of obscuring anthocyanins green chlorophyll (Saniewski et al., 2022).

**Table 2:** The efficiency of inactivation of *FtPDS* gene with CRISPR/Cas9 system using pGG-Cas9-GFP-AtU6-FtPDS-kanR vector.

Observed phenotype	Wild-type	Albino plants	Mosaic plants	Total number of regenerated plants
Number of plants	23	6	1	30
% of all regenerated plants	77%	20%	3%	100%

## 3 Discussion

In this study, we successfully transformed callus derived from immature zygotic embryos of *F. tataricum*. Callus cultures have long been favoured for genetic transformation due to their ease of culture and transformation. Immature zygotic embryos are particularly favourable as the tissue source for callus induction, as they yield stable callus with high regenerative potential (Pratiwi and Surya, 2020). Despite the time-consuming nature of inducing *F. tataricum* callus, which necessitates flowering plants, the resulting callus can be subcultured for extended periods while retaining its genetic stability and morphogenic potential for up to 10 years (Rumyantseva et al., 2003; Rumyantseva et al., 2004; Betekhtin et al., 2017; Betekhtin et al., 2019). The transformation efficiency for the GUS and PDS experiments in *F. tataricum* was approximately 20%. While higher efficiencies have been reported in some species, like cassava and strawberry (Odipio et al., 2017; Wilson et al., 2019), our current protocol enables the generation of multiple transformed plants. We typically regenerate around ten plants from a single transformation that uses approximately 5 g of callus. This allows for successfully generating a few independently transformed plants with 3-4 rounds of transformation. The callus can be easily subcultured and multiplied, providing an ample supply for multiple transformation experiments. Noteworthy, some of the regenerated plants exhibited mosaicism, as observed through GUS staining and inactivation of the *FtPDS* gene. In *F. tataricum* mosaicism can be the result of the regeneration through somatic embryogenesis or organogenesis with both processes happening in regenerating callus cultures. While mosaicism is undesirable (Frank and Chitwood, 2016), most regenerated plants were fully transformed. However, it is crucial to assess the presence of inserts integration or/and CRISPR/Cas9 modifications in the next-generation plants, which are usually used for phenotypic characterisation. Using a visual marker, such as GFP fused with Cas9, can aid in identifying transformed plant parts without needing GUS staining or DNA isolation. Additionally, the regenerating plants can be divided by cutting leaves with meristem allowing for better selection.

Selecting appropriate genetic elements, including promoters, terminators, and codon-optimised gene sequences, is crucial for successful genetic transformation. To ensure flexibility and ease of assembly, the expression vectors employed in this study were constructed using the GreenGate system (Lampropoulos et al., 2013; Decaestecker et al., 2019). All vectors used in this study were designed to confer kanamycin resistance to the transformed plants, as kanamycin has been previously established as an effective selection marker (Mi et al., 2020; Wen et al., 2022). The CaMV35S promoter was utilised to drive the expression of GFP, GUS, and the Cas9-GFP fusion protein, resulting in efficient gene expression. Previous studies have employed the same promoter to generate transgenic hairy roots of *F. tataricum* using *Agrobacterium rhizogenes*-mediated transformation (Wen et al., 2022). In our study, the AtU6-26 promoter was used to drive the expression of the sgRNA targeting the *FtPDS* gene, while the study by Wen et al. (2022) utilised the AtU6-1 promoter. Both promoters demonstrated effectiveness, as evidenced by successfully modifying target sequences. Sequencing analysis of the albino plants revealed single or double nucleotide indels, similar to the modifications observed in the *FtMYB45* gene by Wen et al. (2022). However, authors also observed indels larger than two nucleotides in some cases. Using standard genetic elements commonly employed in dicot genetic engineering appeared suitable for *F. tataricum*. However, future optimisation and utilisation of *Fagopyrum*-specific promoters may offer additional benefits in enhancing genetic manipulation in this species (Zhang et al., 2014; You et al., 2022).

The integration of foreign genetic material, specifically the T-DNA cassette introduced by *A. tumefaciens*, may impose limitations on the usage of transformed plants in field conditions, as they are typically considered genetically modified organisms. Several strategies can be employed to remove the T-DNA cassette. One approach involves crossing the transformed plants with wild-type plants or selecting transgene-free plants in subsequent generations, taking advantage of the self-fertilisation nature of *F. tataricum*. Another strategy involves utilising excision systems, such as the FLP-FRT system (Dalla Costa et al., 2020; Zhang et al., 2021b). Recently, CRISPR ribonucleoprotein-mediated genetic engineering has emerged as a promising alternative method for genome modification (Zhang et al., 2021b). This approach uses Cas9 protein loaded with suitable sgRNA to perform genetic modifications. It offers transgene-free editing with minimal off-target effects, as endogenous proteases and nucleases degrade the ribonucleoprotein complex. This complex can be applied to transfect callus or embryo bombardment, but the most promising approach is protoplast transfection. Notably, an optimized protocol for protoplast regeneration in *F. tataricum* was developed by our group (Zaranek et al., 2023b). We have also recently published an efficient and rapid system of plant regeneration via protoplast cultures of *F. esculentum*, which opens up the possibility of utilizing the ribonucleoprotein complex in these species, while avoiding the issue of mosaicism (Zaranek et al., 2023a). For *F. esculentum*, an alternative approach of transformation is also available. A recently published Agrobacterium-mediated floral dip method, utilising a GFP-expressing vector, achieved a transformation efficiency of up to 12% (Zhou et al., 2023).

While this work represents significant progress in the genetic manipulation of *F. tataricum*, the presented protocol has limitations due to intrinsic differences among cultivars and their ability to form callus. The *F. tataricum* k-17 sample was selected based on previous studies demonstrating efficient callus induction, morphogenic potential, and genetic stability (Betekhtin et al., 2017; Betekhtin et al., 2019). Although the successful transformation of the k-17 sample can be utilised for basic molecular studies and limited field applications, it is necessary to develop a universal protocol for *F. tataricum* that can be implemented across local cultivars to harness the benefits of genetic manipulation fully. Fortunately, the continuous improvement of transformation vectors, such as those containing morphogenic genes like *WUSCHEL2* and *BABYBOOM*, holds promise for enhancing the transformation efficiency of recalcitrant cultivars (Wang et al., 2023).

## 4 Conclusions

This work presents an efficient approach for Agrobacterium-mediated transformation of *F. tataricum* using callus induced from immature embryos as the target tissue. The transformation effectiveness was validated through successful GUS staining, indicating the expression of the introduced genes, and GFP observation, confirming the presence of GFP in the transformed plants. Additionally, the inactivation of the *FtPDS* gene, resulting in albino plants, provided further evidence of successful transformation and gene editing in *F. tataricum*. The results of this study offer a valuable tool for genetic manipulation and trait enhancement in this important crop species.

## 5 Materials and methods

### 5.1 Plant materials and growth conditions

The study utilised the seeds of *F. tataricum* (sample k-17) obtained from the N. I. Vavilov Institute of Plant Genetic Resources collections in Saint Petersburg, Russia. *F. tataricum* sample k-17 represents a commonly cultivated landrace of *F. tataricum*, and seeds can be obtained upon request from the publication’s authors. The plants were grown under field conditions from May to September. The morphogenic calli lines were derived from immature zygotic embryos in the dark at 26 ± 1°C using an RX medium as described previously (Betekhtin et al., 2017; Betekhtin et al., 2019; Zaranek et al., 2023a). The RX medium consisted of B5 mineral salts supplemented with 2.0 mg/L thiamine-HCl, 1.0 mg/L pyridoxine-HCl, 1.0 mg/L nicotinic acid, 2000 mg/L casein hydrolysate, 2.0 mg/L 2.4-D, 0.5 mg/L indolylacetic acid, 0.5 mg/L naphthylacetic acid, 0.2 mg/L kinetin, 2.5% sucrose and 0.8% agar (Gamborg et al., 1968). The morphogenic calli cultures were subcultured every four weeks. A callus in the middle of the passage containing visible PECCs on the surface was used for the morphogenic callus transformation.

### 5.2 PDS sequencing analysis

The partial region of the *PDS* gene in *F. tataricum* (*FtPinG0302920600.01*) was amplified using primers designed based on the *F. tataricum* cv. Pinku1 genome assembly as described by Zhang et al. (2017) (**Table S1**). The amplified PCR product was subsequently cloned into the pGEM-T Easy vector (Promega, USA) and sequenced. The partial sequence of *FtPDS* data have been submitted to the GenBank databases under accession number OR334360. To identify target sequences within the *FtPDS* gene, the Find CRISPR Sites tool in Geneious Prime 2022.0.2 was utilised. The activity of the target sequences was assessed using Doench et al. (2014) scoring method, while specificity was evaluated following the Hsu et al. (2013) criteria. For the alignment of the first exon sequence of the *PDS* gene, the genomes of *F. tataricum* cv. Pinku1, *F. esculentum* cv. DASHA, and the sequencing results for *F. tataricum* sample k-17 were utilized (Zhang et al., 2017; Penin et al., 2021).

### 5.3 Vectors preparation

The vectors for GFP (pGG-CaMV35S-GFP-NLS-kanR) and GUS (pGG-CaMV35S-GUS-kanR) expression were generated using the GreenGate system as described by Lampropoulos et al. (2013). The plasmid kit (GreenGate system) used to generate plant transformation constructs was a gift from Jan Lohmann (Addgene kit # 1000000036). The vectors were assembled using the Golden Gate reaction by ligating vectors: pGGZ004 (Lupanga et al., 2020) (vector backbone conferring spectinomycin resistance), pGGA004 (CaMV35S promoter), pGGB005 (N-tag, B-dummy), pGGD002 (C-tag, B-dummy), pGGE001 (RBCS terminator), pGGF007 (pNOS : KanR:tNOS), and pGGC012 (CDS, GFP with NLS) for GFP expression and pGGC051 (CDS, GUS) for GUS expression. The construction of the pGG-Cas9-GFP-AtU6-lacZ-kanR vector for sgRNA cloning involved several steps. The primers utilised for amplifying the elements required for vector and sgRNA construction are provided in **Table S1**. Firstly, the plant codon-optimised Cas9 gene was amplified from the pYPQ167 vector, and the STOP codon was removed during amplification (Lowder et al., 2015). The amplified Cas9 gene sequence was then cloned into the empty CDS entry vector (pGGC000), resulting in the vector pGG-Cas9(ΔSTOP). For sgRNA cloning, the pGG-F-AtU6-BbsI-ccdB-BbsI-G vector (contains promoter AtU6-26) was modified by removing the ccdB cassette through digestion with the BbsI restriction enzyme (Decaestecker et al., 2019). The resulting linear backbone was ligated with the AarI-lacZ-AarI PCR product, which was digested with the AarI enzyme, creating the pGG-F-AtU6-AarI-lacZ-AarI-G vector. The AarI-lacZ-AarI insert was cloned from vector JD633 (Debernardi et al., 2020). JD633 was a gift from Jorge Dubcovsky (Addgene plasmid # 160393; http://n2t.net/addgene:160393; RRID : Addgene_160393). Finally, the final vector, pGG-Cas9-GFP-AtU6-lacZ-kanR, was assembled by ligating vectors: pGGA004 (CaMV35S promoter), pGGB005 (N-tag, B-dummy), pGG-Cas9(ΔSTOP), pGGD001 (linker-GFP), pGGE001 (RBCS terminator), pGG-F-AtU6-AarI-lacZ-AarI-G (empty vector for cloning sgRNA), and pGGK-AG (vector backbone). The oligos containing the sgRNA sequence and 5’ overlap sequence (5’-ATTG-N20-3’ and 5’-AAAC-N20 (reverse complement)-3’) specific to *FtPDS* were annealed and cloned into the empty gRNA destination vector, pGG-Cas9-GFP-AtU6-lacZ-kanR. This resulted in the generation of the vector pGG-Cas9-GFP-AtU6-FtPDS-kanR. The proper assembly of the donor and final vectors was verified by Sanger sequencing. Subsequently, the vectors pGG-Cas9-GFP-AtU6-FtPDS-kanR, pGG-CaMV35S-GFP-NLS-kanR and pGG-CaMV35S-GUS-kanR were transformed into *A. tumefaciens* GV3101 electrocompetent cells (Gold Biotechnology) and selected on LB plates supplemented with rifampicin (25 μg/mL), spectinomycin (25 μg/mL), and gentamycin (50 μg/mL), as previously described (Hus et al., 2020). For all PCR reactions Q5 High-Fidelity DNA Polymerase was used (New England Biolabs, USA). The detailed schematics and sequences of generated vectors are presented in **Supplementary Figure S2** and **Supplementary Data 2**.

### 5.4 Agrobacterium preparation for transformation

To initiate the Agrobacterium culture, 5 μL of a glycerol stock of *A. tumefaciens* GV3101 transformed with the respective vectors was inoculated into 1 mL of LB medium containing rifampicin (25 μg/mL), spectinomycin (25 μg/mL), and gentamycin (50 μg/mL). The culture was then incubated at 28°C with shaking at 200 rpm for 24 hours. For plating the overnight Agrobacterium culture, 200 μL of the culture was spread onto LB agar plates supplemented with spectinomycin (25 μg/mL), gentamycin (50 μg/mL), and 30 mg/L acetosyringone. For the transformation process, the Agrobacterium cells were scraped from the plates and added to liquid RX medium supplemented with 30 mg/L acetosyringone and 0.1% Pluronic F-68. The OD_600_ of the culture was adjusted to 0.8, and the culture was incubated at 28°C with shaking at 220 rpm for 1 hour before the callus transformation.

### 5.5 Callus transformation

Approximately 5 g of the morphogenic callus were placed in glass Petri dishes (diameter 100 mm). The calli were then inoculated with 30 mL of an Agrobacterium suspension and incubated for 10 minutes. Subsequently, the Agrobacterium liquid was removed. Then callus was transferred on two filter discs (diameter 110 mm) followed by a 15-min desiccation treatment to limit subsequent overgrowth of Agrobacterium and to promote transformation through an osmotic effect. Next, the infected callus was transferred onto two filter discs (diameter 110 mm) placed on 2 ml of liquid RX medium supplemented with 30 mg/L acetosyringone. The plates were sealed with Parafilm and kept in the dark at 26°C for three days. Following the co-cultivation period, the callus was transferred to RX medium containing 50 mg/L kanamycin, 300 mg/L timentine, and 200 mg/L cefotaxime (all antibiotics from Duchefa) and incubated in the dark at 26 ± 1°C for three weeks. After three weeks, the callus was transferred to a fresh medium with the same antibiotic concentrations and cultivated for three more weeks.

### 5.6 Plants regeneration

After six weeks on the medium with antibiotics, only newly emerged PECCs were isolated and transferred onto the regeneration medium. The regeneration medium consisted of MS basal salts supplemented with 50 mg/L kanamycin, 300 mg/L timentine, 200 mg/L cefotaxime, 2.0 mg/L benzylaminopurine, 1.0 mg/L kinetin, 30 g/L sucrose, and 3 g/L gelrite. The cultures were maintained in a growth chamber at 28 ± 1°C under a 16/8 h (light/dark) photoperiod, with a light intensity of 80 µmol m-2 s-1. Subculturing of calli with developing somatic embryos or shoots was performed every three to four weeks. Fully developed shoots were further transferred to an MS medium containing vitamins but without plant growth regulators, supplemented with 25 mg/L kanamycin, 300 mg/L timentine, 200 mg/L cefotaxime, 30 g/L sucrose, and 3 g/L gelrite.

### 5.7 GUS staining

GUS activity in the calli and leaves of *F. tataricum* was monitored throughout the transformation experiments. The GUS assay was performed by incubating the calli and leaves in a histochemical buffer containing 0.1 M sodium phosphate buffer (pH 7.0), 50 mM EDTA, 0.5 mM K_3_Fe(CN)_6_, 0.5 mM K_4_Fe(CN)_6_, 0.1% Triton-X-100, and 1 mg/mL X-gluc (5-bromo-4-chloro-3-indolyl-β-glucuronidase) overnight at 37°C (Jefferson et al., 1987). After incubation, the calli and leaves were washed with deionised water and cleared with 70% ethanol.

### 5.8 Microscopic observations

To assess the presence of the GFP signal, a portion of the fully developed leaf was transferred onto a slide in a drop of distilled water. The sample was then covered with a coverslip. The GFP signal was examined using a Zeiss Axio Imager Z2 microscope with an AxioCam Mrm monochromatic camera (Zeiss). The images were captured using the corresponding software and narrow-band filters designed for GFP visualisation. The bright field images were captured using a KEYENCE VHX-7000 digital microscope (Keyence, Japan).

### 5.9 Detection of CRISPR/Cas9-mediated mutations

The DNA from the leaves of regenerated plants were isolated using the C-TAB isolation protocol as described previously (Hus et al., 2020). The partial sequence of the *FtPDS* gene was amplified from the isolated DNA samples and cloned into the pGEM-T Easy vector. Four to six bacterial colonies were randomly selected for each plant and subjected to sequencing analysis to ensure coverage of both gene copies. The obtained sequencing data were aligned with the reference sequence using Geneious Prime 2022.0.2 software, allowing for the identification of any mutations in the *FtPDS* gene.

## Data availability statement

The datasets presented in this study can be found in online repositories. The names of the repository/repositories and accession number(s) can be found in the article/**Supplementary Material**.

## Author contributions

AP: Conceptualization, Data curation, Formal Analysis, Investigation, Methodology, Software, Supervision, Validation, Visualization, Writing – original draft, Writing – review & editing. AB: Conceptualization, Data curation, Formal Analysis, Funding acquisition, Investigation, Methodology, Project administration, Resources, Software, Supervision, Validation, Visualization, Writing – review & editing.
