# Supplementary material for: Efficient Agrobacterium-mediated transformation and genome editing of Fagopyrum tataricum
Source: Front Plant Sci. 2023 Sep 8;14:1270150. doi: 10.3389/fpls.2023.1270150 (PMC10515086; doi:10.3389/fpls.2023.1270150)
Supplement: Supplementary file 1 [file Image_1.pdf]

frontiers

Supplementary Material

Efficient Agrobacterium-Mediated Transformation and Genome Editing of *Fagopyrum tataricum*

Artur Pinski*, Alexander Betekhtin*

Faculty of Natural Sciences, Institute of Biology, Biotechnology and Environmental Protection, University of Silesia in Katowice, Jagiellonska 28, 40-032 Katowice, Poland

*** Correspondence:**
Artur Pinski (artur.pinski@us.edu.pl), Alexander Betekhtin (alexander.betekhtin@us.edu.pl)

Supplementary Table S1. The primer sequences used in this study along with their corresponding descriptions.

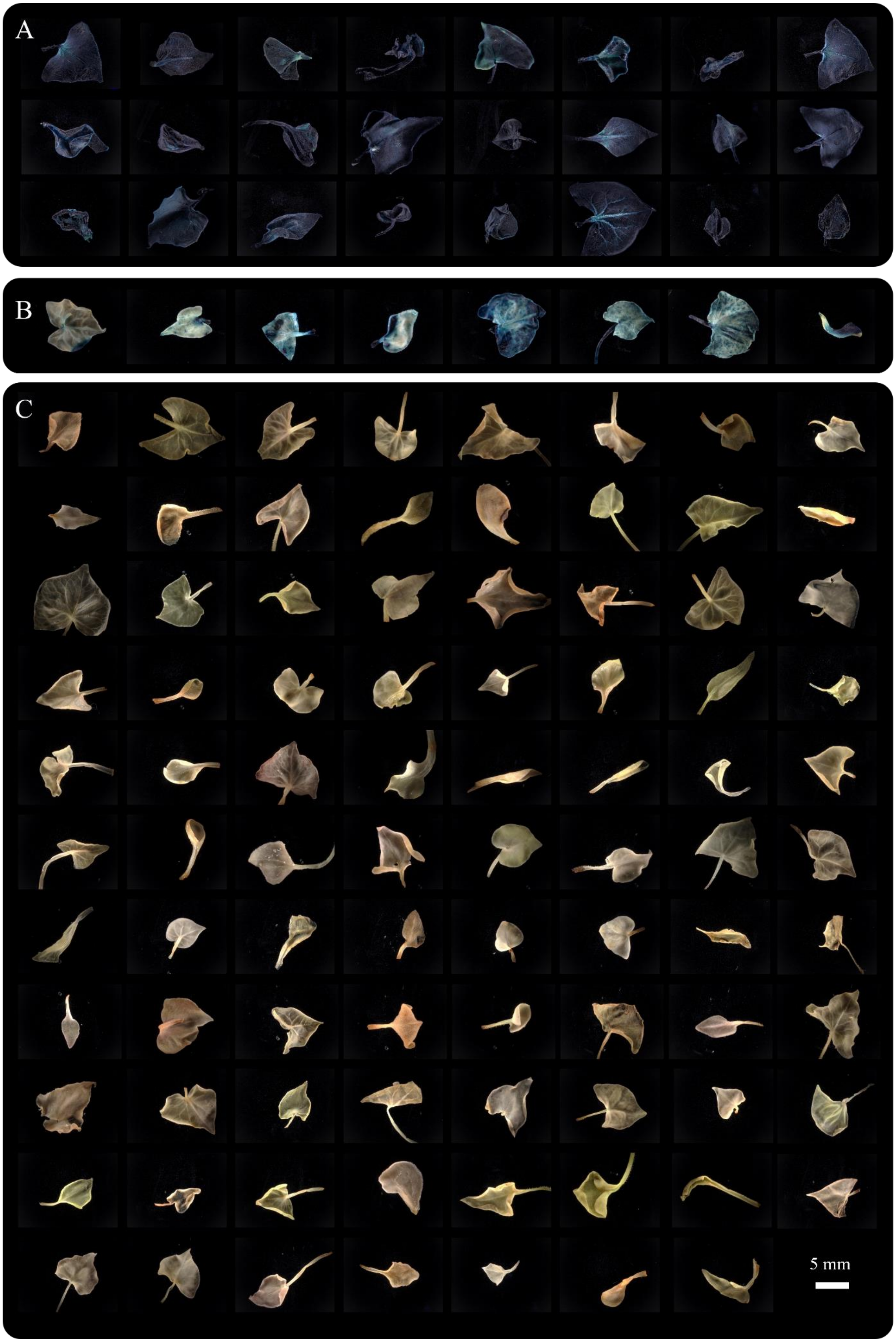
A
B
C
5 mm

Supplementary Figure S1. Visualization of GUS staining of plants of *F. tataricum*. (A) Leaves of transformed plants of *F. tataricum*. (B) Regenerated plant exhibiting mosaicism (C) Leaves of wild-type (WT) regenerated plant of *F. tataricum*.

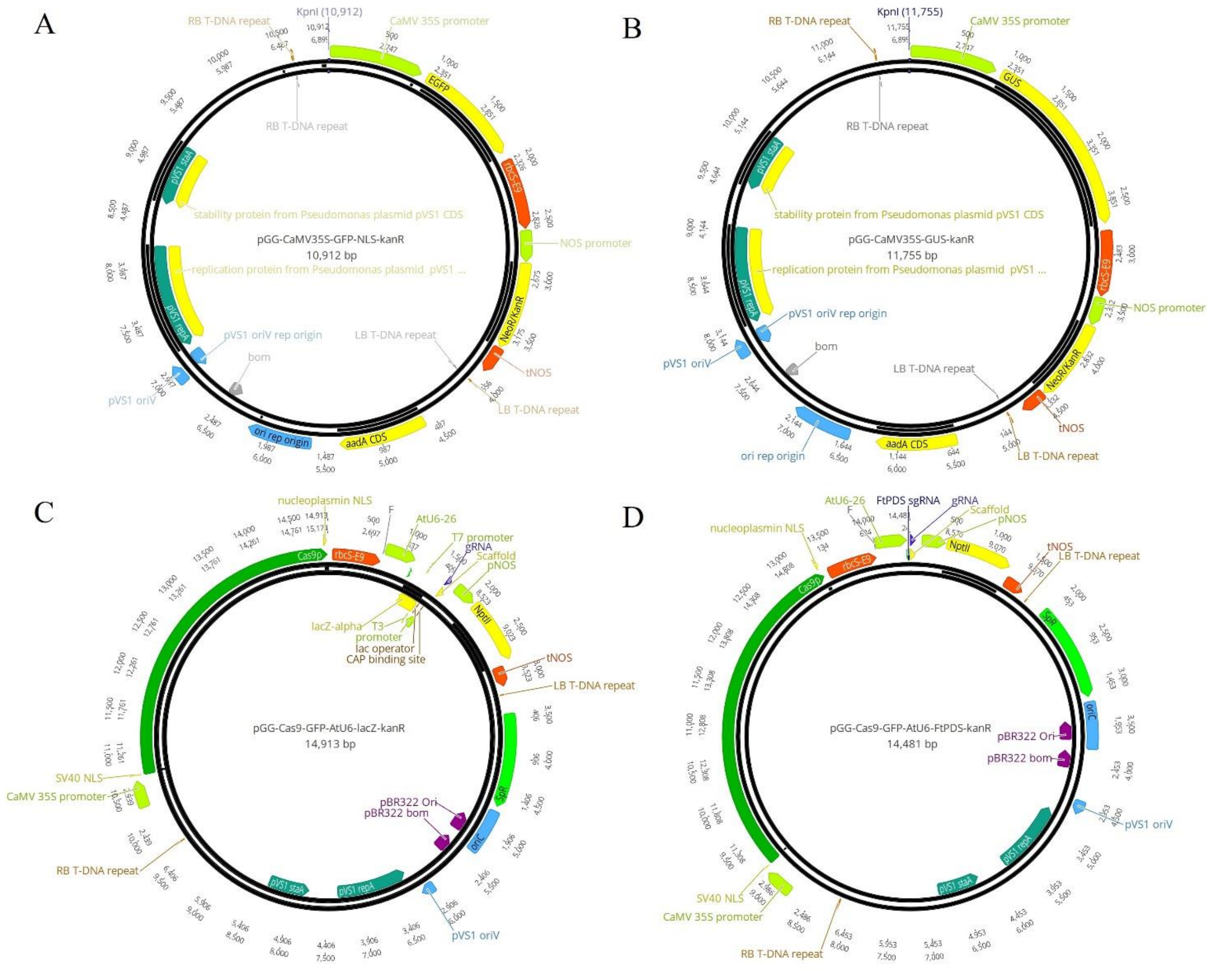

Supplementary Figure S2. The schematic representation of the vectors used in this study: (A) pGG-CaMV35S-GFP-NLS-kanR, (B) pGG-CaMV35S-GUS-kanR, (C) pGG-Cas9-GFP-AtU6-lacZ-kanR, and (D) pGG-Cas9-GFP-AtU6-FtPDS-kanR.

Supplementary Data 1. The unedited chromatograms of the wild-type FtPDS gene and *pds* mutants (ab1 format).

Supplementary Data 2. The sequences of vectors (pGG-CaMV35S-GFP-NLS-kanR, pGG-CaMV35S-GUS-kanR, pGG-Cas9-GFP-AtU6-lacZ-kanR, and pGG-Cas9-GFP-AtU6-FtPDS-kanR) with relevant descriptions (GenBank format).
